# Plant Fungal Diseases and Crop Protection, Second Edition

**DOI:** 10.3390/jof12050372

**Published:** 2026-05-18

**Authors:** Ofir Degani

**Affiliations:** 1 Faculty of Sciences, Tel-Hai University of Kiryat Shmona in the Galilee, Kiryat Shmona 1220800, Israel; ofird@telhai.ac.il or d-ofir@migal.org.il; Tel.: +972-54-678-0114; 2 MIGAL, Galilee Research Institute, Tarshish 2, Kiryat Shmona 1101600, Israel

Fungal pathogens constitute the largest group of plant disease agents, infecting crops through leaves, seeds, and soil, and causing substantial losses in agricultural productivity worldwide. Their diverse infection strategies, together with the complexity of host–pathogen–environment interactions, continue to challenge effective disease management and highlight the need for innovative and sustainable control approaches. Progress in this field depends on a clearer understanding of the fungi involved, the crop developmental stages most susceptible to infection, and the environmental factors that drive disease onset and progression. In parallel, increasing public concern over the extensive use of synthetic chemicals, along with the growing emergence of fungicide-resistant strains, has intensified interest in alternative and environmentally friendly strategies [1,2].

This Special Issue was established to highlight recent advances in the understanding of plant fungal diseases and the development of more effective, integrated, and sustainable crop-protection strategies. The papers brought together in this collection reflect the breadth of the field, spanning antagonistic fungi, biological control, resistance breeding, stress-associated symbioses, fungicide sensitivity monitoring, epigenetic regulation of host susceptibility, and innovative fungicide-delivery systems. Collectively, they illustrate how progress in crop protection increasingly depends on combining fundamental biological insight with practical disease-management tools (Figure 1).

At the microbial resource level, Jamilano-Llames and dela Cruz (Contribution 1) explored endolichenic fungi isolated from the fruticose lichens *Ramalina* and *Usnea*. All isolates suppressed the growth of *Colletotrichum gloeosporioides*, *Cladosporium cladosporioides*, and *Fusarium oxysporum* in dual-culture assays, with direct-contact inhibition identified as the main antagonistic trait. Notably, *Ramalina*-derived isolates generally showed stronger activity. These findings expand the repertoire of fungal antagonists with potential value for sustainable plant disease management.

Complementing this line of work, Mitrović and co-authors (Contribution 2) demonstrated the practical value of *Trichoderma harzianum* K179 in maize, showing in vitro inhibition of the target pathogens *Fusarium graminearum* and *Aspergillus flavus*. Moreover, seed treatment with this biocontrol agent reduced ear rot severity under field conditions, while also improving yield and lowering the concentrations of key mycotoxins, including fumonisins and aflatoxins. Together, these studies highlight the growing importance of biologically based interventions as viable alternatives or complements to synthetic fungicides.

A second major theme of the collection is host resilience, addressed from both breeding and mechanistic perspectives. Hammer et al. (Contribution 3) reported the successful transfer of downy mildew (caused by *Pseudoperonospora cubensis*) resistance from wild Sikkim cucumber accessions into Beit Alpha cucumber types, providing an important advance for a market class in which resistant cultivars have been lacking. Chen et al. (Contribution 4), in turn, demonstrated that inoculation with the root endophytic fungus *Serendipita indica* enhanced the drought tolerance of *Phoebe sheareri* seedlings, an endemic tree species of China, through improved photosynthetic efficiency, reinforced antioxidant defenses, and modulation of hormone synthesis. Although focused on abiotic stress, this work is highly relevant to plant health and crop protection because it highlights the broader role of beneficial fungi in enhancing host vigor and resilience under adverse conditions.

The issue also features important contributions on pathogen monitoring and the molecular basis of fungicide sensitivity. Liu et al. (Contribution 5) established a baseline sensitivity profile for *Stemphylium lycopersici*, the causal agent of tomato gray leaf spot, to the novel succinate dehydrogenase inhibitor (SDHI) fungicide pydiflumetofen. Notably, they identified resistant field isolates in Hebei Province and linked this resistance to mutations in the SDH subunit SdhC, which reduced pydiflumetofen-binding affinity. These findings provide a valuable reference point for resistance surveillance and sustainable fungicide use. At the host-regulatory level, Ge et al. (Contribution 6) showed that the wheat DNA methyltransferase TaMET1 promotes susceptibility to powdery mildew (caused by the biotrophic fungus *Blumeria graminis* f. s. *tritici*) by negatively regulating salicylic acid biosynthesis through repression of the activator gene *TaSARD1*. Their findings place epigenetic control among the important determinants of plant–pathogen compatibility and offer new insight into the molecular regulation of wheat defense.

Finally, the collection highlights innovation in fungicide delivery through the work of Degani et al. (Contribution 7), who developed and evaluated clay-based azoxystrobin formulations for the management of maize late wilt disease caused by *Magnaporthiopsis maydis*. Using bentonite and sepiolite as carriers, the study demonstrated sustained fungicide release, prolonged bioavailability, and preserved antifungal activity, providing proof of concept for a more environmentally compatible and potentially cost-effective delivery strategy. In this way, the paper contributes not only to late wilt management specifically, but also to the broader goal of integrating smarter formulation technologies into sustainable crop protection.

Taken together, the seven papers included in this collection show that contemporary crop protection is moving beyond single-solution approaches. Instead, durable progress is emerging from the integration of biological control, beneficial symbioses, resistance breeding, mechanistic molecular research, resistance monitoring, and advanced formulation technologies. I hope that this Special Issue will serve both as a useful snapshot of current progress and as a stimulus for further research aimed at developing resilient, environmentally responsible strategies for the management of plant fungal diseases in modern agriculture.

## Figures and Tables

**Figure 1:**
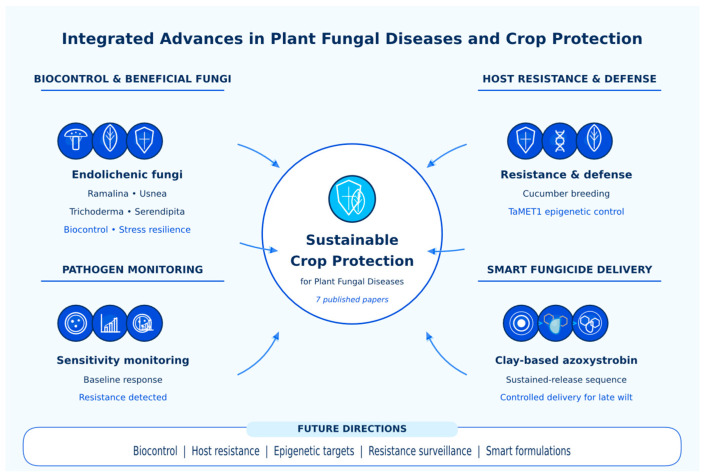
Graphical abstract of the Special Issue Plant Fungal Diseases and Crop Protection, Second Edition. The scheme summarizes the seven papers published in this collection and organizes them into four interconnected themes: biocontrol and beneficial fungi, including endolichenic fungi, *Trichoderma harzianum*, and *Serendipita indica*; host resistance and defense, including the transfer of downy mildew resistance into Beit Alpha cucumber and the epigenetic regulation of wheat powdery mildew susceptibility by TaMET1; pathogen monitoring, represented by baseline sensitivity and resistance detection in *Stemphylium lycopersici*; and smart fungicide delivery, represented by sustained azoxystrobin release from clay carriers for maize late wilt management. Together, these studies highlight the integration of biological control, resistance breeding, molecular regulation, resistance surveillance, and sustainable formulation technologies as complementary pillars of modern crop protection.
